# Psychic Tonus, Body Schema and the Parietal Lobes: A Multiple Lesion Case Analysis

**DOI:** 10.1155/2007/571814

**Published:** 2007-05-30

**Authors:** C. M. J. Braun, S. Desjardins, S. Gaudelet, A. Guimond

**Affiliations:** Centre de Neurosciences de la Cognition and Department of PsychologyUniversité du Québec à MontréalCanada

**Keywords:** Body schema, psychic tonus, hemispheric specialization, parietal lesions, hypergnosia, hypognosia

## Abstract

The psychic tonus model (Braun and colleagues, 1999, 2002, 2003, 2006) states that the left hemisphere is a “booster” of internal experience and behavior in general, and that the right hemisphere is a “dampener”. Twenty-five patients with a “positive” extreme disturbance of body schema (somatoparaphrenia) and 37 patients with a “negative” disturbance of body schema (autotopagnosia or Gerstmann’s syndrome), all following a unilateral parietal lesion, were found in the literature and were analyzed to test predictions from Braun’s “psychic tonus” model. As expected, patients with a positive syndrome had a right hemisphere lesion significantly more frequently, and those with a negative syndrome had a left hemisphere lesion significantly more frequently. Thus the psychic tonus model of hemispheric specialization, previously supported with regard to psychomotor baseline, libido, talkativeness, memory, auditory and visual perceptual tonus, now incorporates the tonus of representation of the body (body schema) in the parietal lobes.

## 1. Introduction

### 1.1. *Psychic tonus: a hemispheric specialization interpretation of positive and negative symptoms*

Braun and colleagues (see Braun [1] for an in depth account) have developed a model of hemispheric specialization initially based on effects of focal lesions which they have termed "psychic tonus". This model proposes a new framework (but see Galin [2] for some premonitory ideas) for interpretation of positive and negative symptoms. The model draws equally from psychiatry as it does from neurology, as well as the biopsychology of normal and abnormal function in humans and animals. The model is based on demonstrations to the effect that mood [3], psychomotor baseline [3], libido [4], talkativeness [5], immune function [6], memory [7], visual symptoms [8] and auditory symptoms [9] are all modulated in similar opposed ways by the two hemispheres, in right handers. The normal left hemisphere increases psychic tonus (infered from right hemisphere lesions), while the right decreases it (infered from left hemisphere lesions). In short, an organism with high psychic tonus will be mentally and behaviorally activated. Even the immune system is upregulated by left hemisphere activation. An organism with low psychic tonus will be all the contrary and will be characterized by right hemisphere activation.

The issue of whether perceptual function also falls under the orbit of hemispheric specialization for psychic tonus remains pending. Braun and colleagues [10] found that there were significantly more reports of cases of post lesion visual hallucination with right hemisphere lesions than left in the behavioral neurology litterature. It therefore appears reasonable to predict that post lesion perceptual anomalies should generally manifest a dissociation according to lesion side. Left hemisphere lesions should produce negative perceptual anomalies, a dulling of perceptual representation which we shall term *hypognosia*, while right hemisphere lesions should produce positive perceptual

ISSN 0953-4180/07/$17.00 © 2007 – IOS Press and the authors. All rights reserved anomalies such as hallucinations, a lushness of perceptual representation, which we shall term *hypergnosia*. More prosaically, “positive” symptoms are understood here to consist of occurrence of undesired, unsollicited representations which ought not occur in a normal subject. We understand “negative” symptoms as inability to mobilize representations which should easily be mobilized at will by a normal person.

One problem with using neurological patients to test the veridicity of the “psychic tonus” concept is that extreme manifestations are required. We acknowledge that such cases are very rare, and that many cases, if not most, may carry bilateral lesions. Pure and extreme perceptual disorders resulting from well documented unilateral brain lesions are too rare to be assembled in a single analysis from any one clinical data base (neurology or psychiatry files of any one institution). That is why the exponentially increasing explosion of case reports today provides a data base fit for inference testing. The present report will limit itself to the somesthesic modality, and to cases with parietal lobe lesions exclusively.

Hemispheric specialization for somesthesic perception has been far less investigated than it has been for vision. Several authors have noted that hysterical somatic symptoms are more typically manifest on the left side of the body (see [11] for a review). This is compatible with the psychic tonus model but it is not the ideal test. As will be explained in the next sections, the ideal test of the psychic tonus model in the somesthesic modality would consist of determining the laterality of focal lesions causing a massively “positive” distorsion of body schema versus a massively “negative’ distorsion. As far as we could determine, body schema has not been subjected to any such analysis crowned by inference tests of lesioned patients designed to test theoretical models of hemispheric specialization.

### *1.2. Psychic tonus and “body schema”*

In their review of post lesion hallucinosis, Braun and colleagues (2003) observed that pure somesthesic hallucination tends to result from right parietal lesions, a finding which has been noted by several predecessors [12]. However, post lesion somesthesic hallucination has been too rare to support inference testing regarding its localisation. Likewise, pure post lesion autotopagnosia or loss of body schema has been noted to nearly always result from left parietal lesions [13,14]. The purpose of the present report was thus primarily to assemble an exhaustive review of single cases with radiological documentation of a focal unilateral parietal lesion leading either to a positive form of distorsion of “body schema” (bilateral somesthesic hallucinosis) or to a negative form (bilateral somesthesic agnosia).

### *1.3. Disorders of body schema*

The term “body image” is often a misnomer in clinical neuroscience. Most clinical neuroscience authors, when talking about “body image” are not talking about body *image* at all, but rather about body *schema*, i.e., of somesthesic representation of one’s own body rather than of visual representation. Though the term body image persists, we contend that it should be replaced by “body schema”. Indeed, the congenitally blind have body schema, i.e., high level representation of body parts. It is significantly less precise than in normals, but it is far from autotopagnosia [15] and this is achieved despite complete absence of visual imagery, even from birth. So obviously visualisation need not be critical in higher order representation of the body. Also, estranged body schema is probably more easily expressed in “visual” terms by patients with expressions such as “I see my body split in two”, “I see a third limb on myself”, etc., when in probable fact, they “feel” those things somesthesically, and translate that feeling into expressions designed to be better understood by the interlocutor. Indeed, when a patient complains of a supernumerary limb, the first comments of the clinician are likely to be: “do you see it? show me where it is . . . etc.”. Suffice it to state that, for the purposes of the present report, we take the term “body schema” to designate a primarily somesthesic high level representation of the body.

The quintessential positive disorder of body schema is somatoparaphrenia [16]. When it is pure and is caused by a unilateral lesion, the localisation of the lesion is typically right parietal (as we shall explicitate in the results section). In the extreme form of somatoparaphrenia complex somesthesic hallucinations involve feelings of supernumerary or abnormally sized or deformed body parts, of feelings of being touched, or of involuntary movement involving both sides of the body. Other terms refering to less obviously “positive” anomalies of body schema include dysmorphobia (or body dysmorphic syndrome), autoscopia, micro and macrosomatognosia, phantom limb, auto-reduplication, self misidentification syndrome (reverse Capgras or Fregoli syndrome, see [17], somatisation disorder, and perhaps heautoscopy and autoscopia though the latter two are visual by definition.

The quintessential and most extreme "negative" disorder of body schema is autotopagnosia [18]. It consists of a complete inability to localize or represent one's own body parts on both sides, a bilateral high level somesthesic agnosia, which often comprises bilateral anosagnosia as well. When it is pure and is caused by a unilateral lesion, the lesion is typically left parietal. Other terms refering to similar but less encompassing negative anomalies of body schema include Gerstmann's syndrome (especially the finger agnosia and right-left confusion elements), asymbolia for pain, anosagnosia, etc. Hécaen and Albert [19] considered Gerstmann's syndrome a mild form of autotopagnosia. Gerstmann's syndrome is relevant to the "psychic tonus" model primarily because one of its symptoms is an intrinsically bilateral distorsion of body schema", namely right-left confusion. We have found that when investigated for such, finger agnosia in Gerstmann's syndrome is also typically bilateral (see the results section), making that syndrome all the more relevant for the present report. Pure Gerstmann's syndrome is nearly always caused by a left parietal lesion. Since many cases of Gerstmann's syndrome have been published, this syndrome was also reviewed systematically for the present report.

### *1.4. Unilateral neglect syndromes and the "psychic tonus" model*

There is overwhelming evidence in behavioral neurology to the effect that unilateral neglect, in any modality – including somesthesic (even with regard to representation of the body), results far more often from right than left lesions, and that the typical lesion producing hemineglect is inferior parietal. We believe that there is probably some truth to this, though exagerated. A pure test of tactile hemineglect must exclude any motor exploration component. Tactile extinction fits that requirement. Gainotti and colleagues [20] found that large cohorts of patients with right and left telencephalic lesions presented no lateralized difference in tactile extinction after correction for errors on the body side ipsilateral to the lesion. Schwartz and colleagues [21] obtained similar results with 274 patients with unilateral brain damage. This explains why many authors may have observed more tactile extinction after right hemisphere lesions: patients with massive left hemisphere lesions tend to get excluded from neuropsychological studies because they are too aphasic to understand elementary instructions, and/or left hemisphere lesioned patients present more bilateral neglect. In short, we believe the so-called "right hemisphere hemineglect" phenomenon is none other than a standard contralesional functional loss which is far less observed in left hemisphere cases simply because the neglect is bilateral in those cases. Nevertheless, the generally recognized propensity of right parietal lesions to produce hemineglect could be construed as the main objection (or perhaps the more exact word would be "challenge") which could be leveled, in the "perceptual domain", against the "psychic tonus" model of hemispheric specialization. Indeed, the psychic tonus model predicts negative cognitive-perceptuomotor symptoms after a left hemisphere lesion, rather than a right hemisphere lesion. Our answer to this is as follows. The dominant models of hemineglect are variants of what could be called the class of "subcortical mediation of orienting". Heilman and colleagues for example, propose a lateralized "corticolimbic reticular loop" and Kinsbourne proposes a "brainstem asymmetry", of lateral orientation [22]. These models insist on primitive pre-representational factors to the detriment of representational factors. One argument for this class of models is that hemineglect is often furtive: it tends to disappear spontaneously after a few weeks after the lesion – suggesting that it is due to a simple primitive mechanism that can be compensated for by representational feedback (the patient learns to invest extra attentional energy to the previously neglected side). Another argument for this class of models is that patients with left hemineglect are hyperoriented to the right side more than they are neglectful of the left side (they look more and they grope more on that side [23]). A third argument in support of this class of models is that subcortical lesions often produce hemineglect, including in the tactile modality [24].

The psychic tonus model has a wider angle than these diverse explanations of hemineglect. It proposes that representational neglect can be bilateral (e.g., autotopagnosia) following a unilateral telencephalic lesion of the left hemisphere, and that the same lesion located in the right hemisphere is more likely to produce the contrary syndrome, a syndrome of pathologically exuberant bilateral representation (e.g., somatoparaphrenia). Since we demonstrate that this is what typically occurs (see the results section), we are led to concur with authors who interpret hemineglect as a pre-representational defect of orientation involving subcortical brain structures. Thus, lesions of the right inferior parietal lobe ought to produce somatoparaphrenia or related symptoms – as well as unilateral left hemineglect, and these two symptom complexes ought to be considered relatively independent – despite the fact that the lesion engendering them can be the same. We agree with Heilman and Kinsbourne that the parietal cortical lesion producing hemineglect probably causes that symptom via loss of connections to subcortical structures, but we add that somatoparaphrenia is a high level representational disorder probably caused directly by damage to parietal cortex. The psychic tonus model proposes that left parietal lesions ought to produce bilateral neglect (of which autotopagnosia is the severest form), without hallucinosis, and as stated above, that is exactly what most often occurs.

Many authors believe that somatoparaphrenia is nothing other than compensation for hemineglect [12, 25–28]. However, somatoparaphrenia is not always a hallucination of an extra body part on the left, it can be on the right body side or both sides or the body meridian, and hemiplegia and hemineglect are not necessarily present (see our results section). Somatoparaphrenia is clearly a "positive" syndrome because it typically consists of spontaneous and exuberant representation of something (e.g., a third limb) that is not in the "normal" repertoire.

## 2. Method

The selection criteria of the cases to be assembled were optimized to assure meaningful interpretation of localisation of brain damage in view of testing the "psychic tonus" model of hemispheric specialization with regard to "body schema". First, to assure that cases with right versus left pathology would be comparable and relevant, only etiologies involving the parietal lobes were considered. Careful attention was paid to lesion localization within the hemisphere to assure against a lesion size artefact. Several other possible intervening variables (age, gender, presence/absence of EEG, presence/absence of neurological exam, date of publication, presence/absence of aphasic symptoms, lesion etiology, presence/absence of hemineglect, psychiatric comorbidity, hemibody where the hallucination occurs) were also systematically tabulated for all cases.

## 3. Results

### *3.1. Positive distorsions of body schema*

The first table reviews published cases of the most extreme form of positive distorsion of body schema resulting from a unilateral parietal lesion, with or without hemineglect. Cases with hemineglect are slightly less telling (for a test of the psychic tonus model of hemispheric specialization) than cases without hemineglect because there probably is indeed a relation between unilateral hallucinosis and unilateral hemineglect – consisting of compensation for sensory deprivation. Such cases present a mixture of positive and negative symptoms – to the extent that hemineglect can be construed as a "negative" symptom (though we think it should not be considered negative psychic tonus). We found only one case presenting both somatoparaphrenia and autotopagnosia and he was excluded because of the presence of both positive and negative body schema symptoms. Other less obviously "positive" bilateral higher order syndromes of distorted body schema (e.g., somatisation disorder, reverse Capgras syndrome) have been reported much less frequently to result from unilateral brain lesions. We found only two cases of reverse Capgras syndrome following a unilateral lesion, and though both had right hemisphere lesions, these were not parietal [29,30]. We found only three cases of somatisation disorder after a unilateral parietal lesion [31–33]. All three had a right hemisphere lesion. However, in the next table, we review only cases with somatoparaphrenia following a unilateral parietal lesion. See Table 1.

Of the 25 cases of somatoparaphrenia following a unilateral parietal lobe lesion of table 1, 24 had a lesion on the right and one on the left (binomial: $p < 0.0005$, two tailed).

We believe left and right hemisphere lesions have equiprobable chances of occuring and being selected for case reports (in the absence of selection for any type of mental disturbance). For example, Montour-Proulx and her colleagues [59] assembled 635 previously published post-lesion cases without selection for any psychiatric symptom. In their data base, 328 cases were left hemisphere damaged and 307 were right hemisphere damaged (binomial probability against equiprobabilty: $p > 0.12$, two tailed).

### *3.2. Negative distorsions of body schema*

The next table reviews published cases of the most prototypically negative and complete variant of distorsion of body schema, namely autotopagnosia -resulting from a unilateral parietal lesion. By definition, all these cases presented bilateral symptoms. Because several authors consider that autotopagnosia is a semantic aphasia [51], cases without aphasia represent a more interesting test of the psychic tonus model of hemi-

Table 1
Cases of somatoparaphrenia following unilateral parietal lesions

| Age at onset, gender and hand preference | Locus of the lesion | Lesion etiology | Somatic and other symptoms | Clinical considerations concerning possible epilepsy | Psychiatric comorbidity | Reference |
|---|---|---|---|---|---|---|
| 72 Female H? | Right temporo-parietal | Infarct | Somatoparaphrenia (supernumerary left limb), left hemineglect | No mention of EEG or seizures | ? | Worthington et al., 1996 [28] |
| 64 Female LH | Right > Left parietal | Meningioma | Somatophrenia (2 legs on each side, uninterrupted for two weeks), slight left visuospatial hemineglect | A few partial seizures of the left leg are mentioned, but EEG is not mentioned | Not confused or disoriented | Vuilleumier et al., 1997 [34] |
| 41 Male RH | Right parieto-temporal | Hematoma | Somatoparaphrenia (supernumerary feet, hands and fingers on the left), left hemineglect that disapeared after 4 months | No mention of EEG or seizures | Impulsivity in keeping with his previous lifestyle, "no evidence of psychotic paranoid ideas or depressive symptoms" | Halligan et al., 1995 [26] |
| 50 Female RH | Right parietal | Hematoma after clipped aneurism due to a arterovenous malformation | Bilateral somatoparaphrenia (macropsia of self and others, misidentification of limb), alien hand syndrome (LH), no hemineglect | No signs of paroxysm in EEG, only slowing over lesion | ? | Leiguardia et al., 1993 [35] |
| 85 Female H? | Right parietal and thalamic | Infarct | Somatoparaphrenia (misidentification of left body), no hemineglect | No mention of seizures or EEG | ? | Paulig et al., 2000 [36] |
| 64 Male H? | Right parieto-temporo-occipital | Infarct | Somatoparaphrenia (left arm is a live baby), no hemineglect | No mention of seizures or EEG | No history of psychiatric illness, agitation and delusions following the stroke, visual and auditory hallucinations | Richardson, 1992 [37] |
| 36 Male RH | Right parieto-occipital | Oligodendroglioma | Somatoparaphrenia (autoscopia in left field), no hemineglect | EEG revealed only slowing over the lesion, no evidence of epilepsy | ? | Maillard et al., 2004 [38] |
| 46 Male RH | Right parietal | Meningioma | Bilateral somatoparaphrenia (his right body slides behind his left, features of the right face are "more prominent" han the left), the devil controls left side, auditory and visual hallucinations, mild left hemineglect | The patient had epilepsy but the somatoparaphrenia was continuous and the interictal EEG was normal | Heavy alcohol use, depression, psychosis and delusions | Nightingale et al., 1982 [39] |

Table 1, continued

| Age at onset, gender and hand preference | Locus of the lesion | Lesion etiology | Somatic and other symptoms | Clinical considerations concerning possible epilepsy | Psychiatric comorbidity | Reference |
|---|---|---|---|---|---|---|
| 69 Female H? | Right parieto-temporo-occipital | Infarct | Somatoparaphrenia (misidentification of sentient left arm and thumb), anosagnosia, left hemineglect | No mention of EEG or seizures | Logorhea | Rode et al., 1992 [27] |
| 63 Male RH | Right parieto-fronto-temporal | Infarct | Bilateral somatoparaphrenia (midline limb), apraxia, spatial agraphia, acalculia, left hemineglect | Right fronto-temporal slowing of EEG | Psychotic episode, delusions, mania, psychosis, confabulation, depression and paranoia | Berthier et al., 1987 [40] |
| 86 Female RH | Right parieto-fronto-temporal | Emboly | Somatoparaphrenia (misidentification of sentient left hand), anosagnosia, left hemineglect | No mention of EEG or seizures | No history of psychiatric illness | Assal, 1983 [41] |
| 84 Female RH | Right fronto-parieto-occipital | Infarct | Somatoparaphrenia (attributes sentient left limb to someone else), visual left hemineglect | No EEG or seizures reported | ? | Bisiach et al., 1991 [42] |
| 77 Female RH | Right fronto-parieto-temporal +putamen and pallidum | Hemorrhage | Somatoparaphrenia (her left sensient hand belongs to someone else), left hemineglect, anosognosia | No EEG or seizures reported | "Oriented in time and space and did not show any other sign of mental deterioration" | Bottini et al., 2002 [12] |
| 85 Female H? | Right parietal and thalamic | Infarct | Somatoparaphrenia (misidentification of left body), left hemineglect | No mention of seizures or EEG | ? | Paulig et al., 2000 [36] |
| 76 Female H? | Right parieto-occipital | Infarct | Somatoparaphrenia (content unspecified), left hemiplegia, left neglect | No mention of EEG or seizures | ? | Ramachandran, 1996 [43] |
| 57 Female H? | Right hemisphere | Several hemorrhages | Somatoparaphrenia (supernumerary hands), no hemineglect | EEG slowing over the right hemisphere, no mention of seizures | Depression | Weinstein et al., 1954 [44] |
| 77 Male RH | Right occipito-parieto-temporal | Infarct | Somatoparaphrenia (supernumerary arms) on the right hemibody, left hemineglect | No mention of EEG or seizures | ? | Sellal, 1996 [45] |
| 70 Female H? | Right parieto-temporal | Atrophy (ganglionic degeneration) | Somatoparaphrenia (misidentification of the left hand), no hemineglect | EEG slowing over the lesion, right hypoperfusion on SPECT, no mention of seizures | ? | Carrilho et al., 2001 [46] |

Table 1, continued

| Age at onset, gender and hand preference | Locus of the lesion | Lesion etiology | Somatic and other symptoms | Clinical considerations concerning possible epilepsy | Psychiatric comorbidity | Reference |
|---|---|---|---|---|---|---|
| 75 Female H? | Right parieto-temporal | Atrophy | Somatoparaphrenia (misidentification of the left hand), no hemineglect | No mention of EEG or seizures | ? | Carrilho et al., 2001 [46] |
| 63 Male H? | Left > Right parietal | Atrophy | Somatoparaphrenia (misidentification of the right hand), right hemineglect | EEG slowing over the lesion, left > right temporo-parietal hypoperfusion on SPECT, no mention of seizures | ? | Carrilho et al., 2001 [46] |
| 60 Female H? | Right parietal | Infarct | Somatoparaphrenia (misidentification of the left hand), no hemineglect | No mention of EEG or seizures | ? | Carrilho et al., 2001 [46] |
| 35 Male H? | Right capsulo-lenticular, parieto-frontal | Hematoma | Somatoparaphrenia (supernumerary left arm or leg), left hemineglect | No mention of EEG or seizures | ? | Donnet et al., 1997 [25] |
| 61 Female H? | Right sub-parietal white matter | Hemorrhage | Somatoparaphrenia (supernumerary left arm), no hemineglect | No mention of EEG or seizures | No delusions or confabulation, reactive depression | Canavero et al., 1999 [47] |
| 36 Male H? | Right parietal | Aneurysm | Somatoparaphrenia (misidentification of the left hemibody, supernumerary legs and arms), no hemineglect | No mention of EEG or seizures | ? | Fredericks, 1963 [48] |
| 26 Male H? | Right parietal | Penetrating wound | Somatoparaphrenia (autoscopia, sensation that his body was dislocated, macroscopia), no hemineglect | No mention of EEG or seizures | No previous experience of depersonnalization or any other psychiatric disturbance | Lunn, 1970 [49] |

Note. None of these cases were reported to have aphasic symptoms.

spheric specialization. Presence of aphasic symptoms was systematically noted. See Table 2.

All four symptoms of Gerstmann's syndrome (agraphia, acalculia, right-left confusion, finger agnosia) were thought by Gerstmann [16] to be high-order disorders of "body image". The rationale for acalculia being a disorder of body schema is that we learn to count with our fingers. The rationale for agraphia being a disorder of body schema is that the agraphia is postulated to be peripheral rather than central, i.e., putatively proprioceptive and/or apraxic. It is frequently argued that Gerstmann's syndrome always comprises and is essentially explainable as a "defective process of mental manipulation of images" [52–55]. Several authors have believed that Gerstmann's syndrome is a form of aphasia [56–58] and one group has even specified that the aphasia is semantic and that the main problem is a breakdown of the part/whole dialectic [59]. Presence of aphasia weakens our test of the psychic tonus model because aphasia falls under the orbit of a form of hemispheric specialization (linguistic) which we fear may be orthogonal to the issue at hand (though we are not sure of that yet). We therefore documented any mention of aphasia. Others have argued that the syndrome does not exist in pure form. However, all the controversy does not detract from the fact that bilateral finger agnosia and right-left confusion (especially relative to self) are obvious defects of body schema of

Table 2
Cases of autotopagnosia or Gerstmann's syndrome following a unilateral parietal lesion

| Age at onset, gender and hand preference | Lesion locus | Etiology | Somatic and other symptoms | Clinical considerations concerning possible epilepsy | Psychiatric comorbidity/ aphasia | Reference |
|---|---|---|---|---|---|---|
| 49 Male H? | Left parietal | Glioma | Autotopagnosia (bilateral), no aphasia | Diffuse slowing of EEG | No mention of psychiatric comorbidity, no aphasia | De Renzi & Faglioni, 1963 [60] |
| 64 Male RH | Left parietal | Tumor | Autotopagnosia (bilateral), no aphasia | No mention of EEG or seizures | No mention of psychiatric comorbidity, no aphasia | De Renzi & Scotti, 1970 [61] |
| 59 Male RH | Left parietal | Cystic tumor (metastatic carcinoma) | Autotopagnosia (bilateral), Gerstmann syndrome, ideomotor apraxia, no aphasia | No mention of EEG or seizures | No mention of psychiatric comorbidity, no aphasia | Ogden, 1985 [62] |
| 67 Male RH | Left parietal | Encephalomalacia | Autotopagnosia (bilateral), dyscalculia, dysgraphia, no aphasia | No mention of EEG or seizures | No mention of psychiatric comorbidity, aphasia | Denes et al., 2000 [13] |
| 74 Female LH | Right parieto-temporo-occipital | Hemorrhagic infarct | Autotopagnosia (bilateral), limb apraxia, neglect syndrome, no aphasia | No mention of EEG or seizures | No mention of psychiatric comorbidity, no aphasia | Verstichel et al., 1994 [63] |
| 64 Female H? | Left parietal | Anaplastic astrocytoma | Autotopagnosia (bilateral), anomia | No mention of EEG or seizures | No mention of psychiatric comorbidity, anomia | Baldini et al., 1978 [5] |
| 56 Male RH | Left parieto-occipital | Hemorrhage | Autotopagnosia (bilateral), mild dysphasia | Left EEG slowing without epileptiform signs | no mention of psychiatric comorbidity, mild dysphasia | Poncet et al., 1971 [59] |
| 71 Female LH | Right parietal (right hemisphere dominant for language) | Encephalomalacia | Autotopagnosia (bilateral), aphasia | No mention of EEG or seizures | No mention of psychiatric comorbidity, aphasia | Denes et al., 2000 [13] |
| 77 Female RH | Left parieto-temporo-occipital | Infarct | Autotopagnosia (bilateral), ideomotor apraxia, aphasia | No mention of EEG or seizures | No mention of psychiatric comorbidity, aphasia | Schwoebel et al., 2001 [14] |
| 74 Female RH | Left parieto-occipital | Metastasic melanoma | Autotopagnosia (bilateral), mild aphasia | No mention of EEG or seizures | No mention of psychiatric comorbidity, mild aphasia | Semenza, 1988 [64] |
| 71 Male H? | Left parieto-occipital | Tissue softening | Complete autotopagnosia with consequent Gerstmann' syndrome | Normal neurological exam, no mention of imagery | No mention of psychiatric comorbidity, no aphasia | Nielsen, 1946 [65] |
| 59 Male RH | Left parietal | Stroke | Gerstmann's syndrome, (bilateral finger agnosia documented), no aphasia | No mention of EEG or seizures | No mention of psychiatric comorbidity, mild word finding difficulties | Varney, 1984 [66] |

Table 2, continued

| Age at onset, gender and hand preference | Locus of the lesion | Lesion etiology | Somatic and other symptoms | Clinical considerations concerning possible epilepsy | Psychiatric comorbidity | Reference |
|---|---|---|---|---|---|---|
| 48 Female H? | Left parieto-occipital | Tumor (multiform spongioblastoma) | Gerstmann's syndrome (bilateral finger agnosia documented), no aphasia | No mention of EEG, seizures or psychiatric comorbidity | No mention of psychiatric comorbidity, no aphasia | Arbuse, 1947 [67] |
| 60 Female H? | Left parieto-occipital | Infarct | Gerstmann's syndrome (bilateral finger agnosia documented), no aphasia | No paroxysms in EEG, slowing over lesion | No mention of psychiatric comorbidity, no aphasia | Perez-Blanco et al., 1989 [68] |
| 64 Male RH | Right parietal | Infarct | Gerstmann's syndrome, (bilateral finger agnosia documented), no aphasia | No mention of EEG or seizures | No mention of psychiatric comorbidity, no aphasia | Calvo-Romero, 2000 [69] |
| 71 Female RH | Left fronto-parietal | Hematoma | Gerstmann's syndrome, (bilateral finger agnosia documented), no aphasia | No mention of EEG or seizures | No mention of psychiatric comorbidity, mild word finding impairment, paraphasic errors | Maeshima et al., 1998 [70] |
| 85 Male ambidextrous | Left parietal | Ischemic stroke | Gerstmann's syndrome (bilateral finger agnosia documented), no aphasia | No mention of EEG or seizures | No mention of psychiatric comorbidity, no aphasia | Carota et al., 2004 [52] |
| 57 Female LH | Right parietal | Stroke | Gerstmann's syndrome, (bilateral finger agnosia documented), dysphasia | No mention of EEG or seizures | No mention of psychiatric comorbidity, dysphasia | Moore et al., 1991 [71] |
| 69 Male ambidextrous | Left parieto-occipital | Hemorrhage | Gerstmann's syndrome (bilateral finger agnosia documented), no aphasia | No mention of EEG or seizures | No mention of psychiatric comorbidity, no aphasia | Dozono et al., 1997 [72] |
| 52 Male RH | Left parietal | Stroke | Gerstmann's syndrome (bilateral finger agnosia documented), no aphasia | No EEG or seizures reported | Euphoria, no aphasia | Sobota et al., 1985 [73] |
| 44 Male RH | Left parietal | Penetrating head injury | Gerstmann's syndrome (bilateral finger agnosia documented), no aphasia | No mention of EEG or seizures | No mention of psychiatric comorbidity, no aphasia | Mazzoni et al., 1990 [74] |
| 59 Male RH | Left parietal | Infarct | Gerstmann's syndrome (bilateral finger agnosia documented), impairment of visual rotation, no apraxia or aphasia | No mention of EEG or seizures | No mention of psychiatric comorbidity, no aphasia | Mayer et al., 1999 [75] |
| 72 Female RH | Left parietal | Glioblastoma multiforme | Gerstmann's syndrome with bilateral toe agnosia (bilateral finger agnosia documented), no aphasia | No mention of seizures or EEG | No history of psychiatric illness, no aphasia | Tucha et al., 1997 [76] |

Table 2, continued

| Age at onset, gender and hand preference | Locus of the lesion | Lesion etiology | Somatic and other symptoms | Clinical considerations concerning possible epilepsy | Psychiatric comorbidity | Reference |
|---|---|---|---|---|---|---|
| 58<br>Male<br>RH | Left parietal | Stroke | Gerstmann's syndrome, (bilateral finger agnosia documented), semantic dysphasia | No mention of EEG or seizures | No mention of psychiatric comorbidity, semantic dysphasia | Ardila et al., 2000 [56] |
| 7<br>Male<br>H? | Left parietal | Infarct | Gerstmann's syndrome, no aphasia | No mention of EEG or seizures | No mention of psychiatric comorbidity, no aphasia | Ohtagaki et al., 1998 [77] |
| 12<br>Male<br>H? | Left parietal | Atrophy | Gerstmann's syndrome, no aphasia | No mention of EEG or seizures | No mention of psychiatric comorbidity, no aphasia | Garty et al., 1989 [78] |
| 64<br>Male<br>RH | Left parietal (angular and supramarginal gyri) | Infarct | Gerstmann's syndrome, no aphasia | No mention of EEG or seizures | No mention of psychiatric comorbidity, no aphasia | Roeltgen et al., 1983 [79] |
| 65<br>Male<br>RH | Left parieto-occipital | Infarct | Gerstmann's syndrome without alexia, apraxia or aphasia | No mention of EEG or seizures | No mention of psychiatric comorbidity, word-finding difficulty | Levine et al., 1988 [57] |
| 56<br>Male<br>RH | Left parieto-temporal | Infarct | Gerstmann's syndrome, no aphasia | EEG slowing over lesion, no mention of seizures | No mention of psychiatric comorbidity, no aphasia | Kinsbourne et al., 1974 [80] |
| 47<br>Male<br>RH | Left parietal | Astrocytic glioma | Gerstmann's syndrome, dysphasia | No mention of EEG or seizures | No mention of psychiatric comorbidity, dysphasia | Kinsbourne et al., 1962 [81] |
| 65<br>Male<br>LH | Left parieto-occipital | Meningioma | Gerstmann's syndrome, no aphasia | EEG slowing over lesion, no mention of seizures | No mention of psychiatric comorbidity, no aphasia | Kinsbourne, 1962 [81] |
| 7<br>Male<br>RH | Left parieto-occipital softening | Infarct | Gerstmann's syndrome, ADHD, learning disability, no aphasia | EEG was normal bilaterally, no mention of seizures | No mention of psychiatric comorbidity, no aphasia | Fournier-Del Castillo et al., 2000 [82] |
| 67<br>Male<br>RH | Left parietal | Stroke | Gerstmann's syndrome, aphasia | No mention of EEG or seizures | No mention of psychiatric comorbidity, aphasia | Sheimo et al., 1997 [58] |
| 65<br>Male<br>RH | Left parietal | Haematoma | Gerstmann's syndrome, no aphasia | No mention of EEG or seizures | No mention of psychiatric comorbidity, no aphasia | Trillet et al., 1989 [83] |
| 52<br>Male<br>RH | Left parietal | Haematoma | Gerstmann's syndrome, no aphasia | No mention of EEG or seizures | No mention of psychiatric comorbidity, no aphasia | Trillet et al., 1989 [83] |

Table 2, continued

| Age at onset, gender and hand preference | Locus of the lesion | Lesion etiology | Somatic and other symptoms | Clinical considerations concerning possible epilepsy | Psychiatric comorbidity | Reference |
|---|---|---|---|---|---|---|
| 68 Male LH | Right parietal | Infarct | Gerstmann's syndrome, no aphasia | No mention of EEG or seizures | No mention of psychiatric comorbidity, no aphasia | Dehaene et al., 1997 [84] |
| 79 Male RH | Left parietal | Infarct | Gerstmann's syndrome, no aphasia | No mention of EEG or seizures | No mention of psychiatric comorbidity, no aphasia | Gold et al., 1995 [53] |

the agnosic type (though manipulation of images may be a problem as well, and the problem can be verbally mediated), and these two symptoms affect both sides of the body, making them highly relevant tests of the "psychic tonus" model. At any rate, the controversy over Gerstmann's syndrome has resulted in a large set of case reports, useful for testing the psychic tonus model.

Cases of Gerstmann's syndrome following a unilateral parietal lesion which are better suited to testing the psychic tonus model are those where the finger agnosia is documented as bilateral. In Table 3, we noted whether the cases were documented for bilateral agnosia or not. See Table 2.

Of the 37 cases of Table 2, all with a negative disorder of body schema, 32 had a left hemisphere lesion and 5 had a right hemisphere lesion (binomial: $p < 0.0005$, two tailed).

Any issue of a general bias in published case reports for lesions in one or the other hemisphere is eschewed by a test of the prediction of a crossed double dissociation. All the post lesion cases with positive syndromes (somatoparaphrenia) ($N = 25$) were therefore compared to all the post lesion cases with a negative syndrome (autotopagnosia, Gerstmann's syndrome) ($N = 37$). The crossed double dissociation between lesion side and type of somesthesic disorder is highly significant ($\mathrm{Chi}^2 = 40.77$, $p < 0.0005$, two tailed).

### 3.3. *Secondary analyses for purposes of artefact control (all cases, $N = 62$)*

The main results reported above appear to be highly significant and thus, to be concordant with the psychic tonus model. However, it is important to determine whether that inference test withstands challenges from eventual contamination from extraneous sampling bias.

The analyses to be presented next comprise tests of alternative explanations of the interaction between somesthesic disorder type and lesion side. We operationalized this interaction as a dichotomous variable which we termed "concordance with the psychic tonus model". A left lesion associated with autotopagnosia\Gerstmann syndrome or a right lesion associated with somatoparaphrenia was rated as 1, the other eventualities were rated as 2. In the case of a significant relation between "concordance with the psychic tonus model" and an extraneous variable, a second phase of analysis was to be implemented, subjecting the theorical effect to partial correlation – retrenching the variance of the contaminant. This analytical framework supposes that only dichotomous variables may be processed. Consequently all control variables were to be eventually dichotomized if not naturally limited to two levels.

### 3.4. *Hand writing preference*

Hand writing preference is an important variable because non-right handers present reversed hemispheric specialization in 10 to 30% of cases. Biased sampling of hand preference could thus bring a caveat to the main result (concordance with the psychic tonus model by lesion side). In this study, there were 32 right handers of which 31 were concordant with the psychic tonus model and 8 left handers or ambidextrals or which 4 were concordant with the psychic tonus model (the other cases' hand preference was not reported). The relation between these two variables is significant, the non-right handers being less concordant with the psychic tonus model (50%) than the right handers (96.9%). A partial correlation was therefore computed between lesion side and type of somesthesic disorder, controlling for hand writing preference. The psychic tonus effect remained significant despite statistical removal of the effect of handedness ($rp = 0.82$, $p < 0.0005$).

### 3.5. Other control variables

Locus of the lesion was analyzed because though parietal lobe lesions were a selection criterion, damage elsewhere was not an exclusion criterion, and thus an extra parietal lesion sampling artefact had to be ruled out. Lesion size was analyzed because larger lesions can be thought to produce loss of mental function more often than smaller lesions, and thus a sampling bias could contribute an artefact. Etiology of the lesion was of interest because some etiologies, like tumor, can sometimes irritate and excite brain tissue and create cognitive symptoms [85]. Head trauma can also introduce noise into the data base because it presents with diffuse bilateral damage that is too subtle to be radiologically localized. Cases with old (ex: congenital) lesions could present far more cognitive compensation, adding noise to the inference tests. Presence of a psychiatric comorbidity was analyzed because somatic hallucination or delusion, though less frequent than auditory or visual hallucination, are not rare in psychotics [86,87]. The age of the patient is of interest because a bilateral diffuse stress on the brain is associated with aging. Juvenile cases also present less hemispheric specialization than adults. Gender was of interest because women tend to have less focal cognitive representation in the hemispheres and also less functional lateralization. The date of publication is pertinent because MRI technology used nowadays as a diagnostic method is more precise than CT scan. The presence of an EEG was of concern because it brings more precision to lesion localization and reduces chances of contamination by epileptiform activity. The presence/absence of aphasic symptoms is an important control variable because of the well known hemispheric specialization for linguistic abilities (see [88] for a review). However, none of these variables was significantly related to "concordance with the psychic tonus model".

### 3.6. More specific analyses (somatoparaphrenic group only, $N = 25$)

In the neurological literature, it is well known that positive symptoms often occur in the contralateral or the neglected hemibody [89]. In the current study, 20 cases out of 25 had their target symptoms occuring in the hemibody contralateral to the lesion ($\text{Chi}^2 = 7.64$, $p = 0.054$). There are however four cases of right hemisphere lesions, thus concordant with the psychic tonus model, for whom the symptoms occured bilaterally. Power was lacking to measure statistically the psychic tonus model inference with these cases only. See Table 3.

Table 3
Distribution of symptom lateralization

| Hemibody where the target symptom occured | | | |
|---|---|---|---|
| Right | Left | Bilateral | Unspecified |
| 3 | 17 | 4 | 1 |

In the current data base, there were 12 non neglecting cases and 13 cases of hemineglect. This variable was not related to lesion side and was thus given no further consideration.

## 4. Discussion

The results of this investigation strongly support the psychic tonus model -extending it to somesthesic perception in the parietal lobes. Unilateral parietal lesions typically cause a positive disturbance of body schema when they are located in the right hemisphere and a negative disturbance of body schema when they are located in the left hemisphere.

Why would a focal left parietal lesion, and only that lesion, impair a patient's finger gnosis (mental representation of the finger) at both hands as it does in Gerstmann's syndrome? Primary sensory somesthesic representation has been mapped in many species and has always been found to be fully contralateral and to present the same topography in each hemisphere in each posterolandic gyrus. None of the published reports on Gerstmann's syndrome has addressed the question of why both hands should manifest finger agnosia -except with perplexity [73] and when the bimanual nature of the agnosia is revealed, it is mentioned only incidentally or obliquely. The fact that autotopagnosia [62,90,91] or Gerstmann's syndrome is so often due to a left hemisphere lesion has inspired several authors (e.g., [67, 74]) to state that body schema is left-hemisphere specialized – a thesis we categorically reject. Likewise, the fact that somatoparaphrenia can be due to a right sided lesion has led several other authors (e.g., [47, 92]) to conclude that body schema is right-hemisphere specialized – a thesis we also categorically reject. The laterality of lesions causing disorders of body schema is often acknowledged and stated to be perplexing by many authors not willing to commit themselves to a single hemispheric repository for body schema. Our psychic tonus model provides a clear explanation of the laterality of the lesion findings and the bilaterality of symptoms in the various syndromes of higher order body schema -and will hopefully put an end to some of these expressions of perplexity.

It seems to be a fact that when parietal neocortex is damaged unilaterally, control circuits of psychic tonus can become skewed, and a corresponding disorder of body schema can ensue, positive or negative, as a function of which hemisphere is damaged. Similar phenomena are observed for the other lobes and their respective functions: visual for the occipital lobes [8], auditory, sexual and paralinguistic for the temporal lobes [4,5,7,9], psychomotor for the frontal lobes [3]. These phenomena form an ensemble, a behavioral, motivational and attitudinal system which boils down to a certain type of energy expenditure/economy or approach/avoidance dynamic. Other metaphors of psychic tonus might include contrasts such as "upbeat/downtrodden" or "galavanised/inhibited". For the moment, our favorite designation of psychic tonus is to view it as a mechanism of energy management [1, 93]. In normal behavior, the interplay of these opposed hemispheric mechanisms is finely tuned to situational demands – though we suspect that it oscillates slowly (recall that the immune response is involved, and we expect that various hormones and neurotransmitters are involved as well). However, after a unilateral lesion, the patient becomes set in a mode which is no longer delicately adaptive.

The findings presented here demonstate that localized lesions can produce fractional effects on psychic tonus, such that only one representational modality may be affected – in the present case, somesthesic. Overall, the elements of psychic tonus tend to be correlated in normal and pathological conditions. Thus after a right hemisphere lesion, if a patient becomes manic, he/she (like any congenital manic) is also likely to be (but not necessarily) agitated, talkative, hypersexual, immunofacilitated, delusional and/or hallucinated. After a left hemisphere lesion, if a patient becomes depressed, he/she (like any congenital depressive) is likely to be (but not necessarily) lethargic, hypolalic, hyposexual, immunosuppressed and/or agnosic (see [1,3–5, 7–10] for reviews of the evidence).

It could be thought that the main mediator of the post lesion sticky switch is one or several neurotransmitters. Three good candidates are central norepinephrine serotonin and dopamine (all known to modulate functions subsumed under psychic tonus). Robinson and colleagues [94] ligated the middle cerebral artery of rats. Assays of brain catecholamines revealed 30 percent reductions of norepinephrine in the injured and uninjured cortex and locus coeruleus and a 20 percent reduction of dopamine in the substantia nigra in the right lesioned rats who had also become hyperactive. In contrast rats with left middle cerebral artery ligations did not become hyperactive and did not show any significant change in catecholamines in any of the brain areas studied. Similar asymmetry occurs in serotonin concentrations as a function of stroke side in humans [95]. It remains to be determined whether the latter effect is hemispherically symmetrical or not (a PET ligand study). In fact however we believe that such a neurotransmitter-mediated mechanism is not the principal determinant of hemispheric specialization for psychic tonus effects reported here and elsewhere. Indeed we have found that in non lesional epileptics with unilateral foci ictal hallucination is highly significantly more often observed in cases with left than right foci [96]. This corroboration of hemispheric specialization for psychic tonus cannot be explained as a chronic and major change of neurotransmitter concentration in the brain.

Dominant contemporary methodology in cognitive neuroscience has perhaps not been fully prepared to unveil such mechanisms. Indeed, fMRI cannot be expected (at least at present and without specific precautions) to reveal clearly and unequivocally lateralized (hemispherically specialized) processing, in the normal brain, of higher order body schema, and indeed it generally does not, for example when movement of one limb is required to be imagined [97,98]. Normal high level representation of the body (or of any other cognitive or perceptual function) comprises a balance of positive and negative representation, solliciting both hemispheres for the typical task, thus activating both hemispheres (but each in different aspects of the processing: for example one hemisphere might prepare the movement and the other might inhibit the contralateral mirror movement). A normally optimized high level representation, requiring imagination, polysensory integration, sensorimotor integration, etc., must balance dispositions toward too much imagination (overshooting, tangentiality, profuseness, fabulation, hallucination, etc.) against dispositions toward too much conservatism (inhibition, paucity, inattention, apathy, sluggishness, agnosia, etc.). A typical fMRI experiment subtracts one task from another in view of isolating a specific cognitive operation, thereby canceling out psychic tonus. Most importantly, metabolic imaging studies of cases of extreme distorsion of psychic tonus, specifically of body schema, do not form a corpus that can put a hemispheric specialization model to test. For intimate, subjective, complex experience such as the "body schema" the lesion method can provide insights for behavioral neuroscience where more expensive and prized methods such as metabolic brain imaging need to refine the tasks used in their protocols. With regard to body schema, one intriguing fMRI study required normal subjects to distinguish self movement from movement of another person. This task activated the right parietal lobe, but no theoretical explanation was provided for the laterality of the effect [99]. The "psychic tonus" model would interpret the task as a requirement for inhibition of body schema. Another intriguing fMRI study required normal subjects to localize their own body parts. This activated the left parietal lobe [89]. The "psychic tonus" model would construe this task as a requirement for "activation" of one's body schema. However, distinguishing other people's movement from our own might call upon "inhibition" of one's body schema.

## Acknowledgments

This research was made possible by a grant from the Fonds de Recherche en Santé du Québec (FRSQ) to the first author and a bursary from the Natural Sciences and Engineering Research Council (NSERC) of Canada to the second author.
